# Newly Diagnosed Diabetes and Stress Glycaemia and Its’ Association with Acute Coronary Syndrome

**DOI:** 10.3889/oamjms.2015.103

**Published:** 2015-09-30

**Authors:** Gordana Kamceva, Marija Vavlukis, Darko Kitanoski, Sashko Kedev

**Affiliations:** 1 *University “Goce Delchev”, Faculty of Medical Sciences, Clinical Hospital Shtip, Shtip, Republic of Macedonia*; 2 *Ss Cyril and Methodius University of Skopje, Faculty of Medicine, University Clinic for Cardiology, Skopje, Republic of Macedonia*

**Keywords:** diabetes mellitus, stress glycaemya, hemoglobin A1C, acute coronary syndrome, cardiac events

## Abstract

**BACKGROUND:**

Diabetes is diagnosed in 10-20% of patients with acute coronary syndrome (ACS) not known to be diabetics. Elevated blood glucose is an independent risk factor for cardiac events, regardless of presence of diabetes.

**AIM:**

Evaluating the prevalence of new-diagnosed DM among patients with ACS, and assessing the relationship between stress glycaemia and new diagnosed DM with in-hospital cardiac events.

**METHODS:**

Prospective observational study, in patients with ACS, in whom we analyzed parameters of glycemic metabolism, clinical data, and in-hospital cardiac events. We comparatively analyzed patients according to the HgbA1C and known DM in five groups: non-DM (< 5.6%), new pre-DM (5.6-6.5%), new DM (≥ 6.5%), controlled (<7%) and uncontrolled (≥7%) known DM.

**RESULTS:**

150 patients, (93 male and 57 female) were included. Impaired glucose metabolism was detected in 44.5% of patients, 7.9% of whom were newly-diagnosed DM. The highest levels of stress glycaemia were found in new and uncontrolled known DM. The in-hospital event rate was 20.7%, the mortality rate 7.3%, being the highest in new diagnosed and uncontrolled known DM patients.

**CONCLUSIONS:**

The prevalence of unknown DM was high among patients with ACS. Stress glycaemia and failure to achieve glycemic controlee, were an independent predictors of in-hospital cardiac events.

## Introduction

Diabetes mellitus is increasing on a global level with an estimated prevalence around 12-14%. What is even more important, it is also estimated that one in every four hospitalized patients has known diabetes. Hyperglycemia in hospitalized patients is even more frequent [1].

The Study of Abdullatef et al. performed on Qatar population with acute coronary syndrome (ACS), refers that 45% of hospitalized patients without known DM were either with prediabetes, diabetes or stress hyperglycemia [2]. There are three possible causes for hyperglycemia in hospitalized patients: existing known diabetes, existing but unknown diabetes and stress hyperglycemia. Stress hyperglycemia is defined by ADA (American Diabetes Association) as an elevation of fasting glucose ≥ 7 mmol/L, or 2-hour postprandial glucose ≥ 11 mmol/L, in a patient without evidence of previous diabetes. Glycosylated hemoglobin (HbA1c) value has been recommended to distinguish between patients with stress hyperglycemia and those with previously undiagnosed diabetes. HbA1c value ≥ 6.5% indicates pre-existing unrecognized diabetes, whereas HbA1c value < 6.5% indicates stress-induced hyperglycemia. The prevalence of stress hyperglycemia in critically ill patients varies between 30-40%, 10-15% of which have previously unrecognized diabetes. (3) Gornik et al. reported a prevalence of unrecognized diabetes among critically ill patients of 17% [4]. In the population of elderly patients hospitalized due to heart failure the prevalence of hyperglycemia was 44%, and 41% in patients with acute coronary syndrome [5].

Stress hyperglycemia has several means. Stress conditions such as surgery, trauma and acute illness increase the circulatory level of counter regulatory hormones (glucagon, cortisol, catecholamines) and pro-inflammatory cytokines and they alter the effect of insulin on the hepar and on the skeletal muscle by increasing of the hepatic production of glucose and decreasing the peripheral utilization of glucose. Pro-inflammatory cytokines also increase the hepatic release of glucose and increase the insulin resistance in the hepar and in the skeletal muscle. Stress hyperglycemia in patients with diabetes type 2 involves a combination of insulin resistance and beta cell secretory defect [3].

### The impact of hyperglycemia

Patients with stress hyperglycemia have a higher mortality rate and longer hospitalization time in comparison with patients with known diabetes and with normoglycaemia. They have worse outcome in comparison with diabetic patients with a comparable degree of hyperglycemia. It depends on the underlying diagnose, risk of infection etc. Non-diabetic patients with stress hyperglycemia have 3.9 fold higher risk of death after myocardial infarction in comparison with normoglycaemic non-DM patients. The same finding is also evident in patients with stroke. Worse clinical status is evident in non-diabetic patients with stress hyperglycemia in comparison with diabetic patients [3, 6, 7].

The aim of this study was to evaluate the prevalence of new-diagnosed DM among patients with ACS, and assessing the relationship between stress glycaemia and new diagnosed DM with in-hospital cardiac events.

## Material and Methods

This was a prospective observational study. Patients admitted to ICCU and treated for acute coronary syndrome-ACS (unstable angina-APNS, NSTEMI-myocardial infarction without ST-segment elevation and STEMI-myocardial infarction with ST-segment elevation), were enrolled. All patients with confirmed ACS during the two month period were included. We analyzed glycemic parameters: blood glycaemia at admition (stress glycaemia), fasting plasma glycaemia the first morning after admition, glycaemia levels during the hospital treatment and HgbA1C.

Demographic, clinical, left ventricular functional and angiographic data were obtained for all 150 patients (pts.). We analyzed: body presence of risk factors and co-morbidities, basic biochemical variables (Hgb, BUN, creatinine, Na, K), lipid profile (Tg, HDL, LDL Hol, lpa), LV systolic and diastolic function, SINTAX score, TIMI flow before and after PCI procedure, duration of hospitalization (days) and in-hospital morbidity/mortality: heart failure, malignant arrhythmias, early ischemic events, bleeding complications (CE) and cardiac death (CD).

We used ADA (American Diabetes Association) 2015 Guidelines criteria for the diabetes definition (fasting plasma glycose (FPG) >7 mmoll/L, or random plasma glucose (RPG) >11.1 mmol/L, or HgbA1C >6.5%), and HgbA1C >5.6% for the definition of pre-diabetes; for the definition of stress hyperglycemia: an elevation of FPG ≥7 mmol/L, or RPG ≥ 11 mmol/L in a patient without evidence of previous diabetes. We used glycosylated hemoglobin (HbA1c) value to distinguish between patients with stress hyperglycemia and those with previously undiagnosed diabetes (an HbA1c value ≥ 6.5% indicated pre-existing unrecognized diabetes, whereas HbA1c value < 6.5% indicated stress-induced hyperglycemia). Also, we used the ADA recommendations for controlled diabetes (HgbA1C <7%), to distinguish between diabetic patients with controlled and uncontrolled diabetes. We also used the ADA glycemic target for critically ill patients (6.1-10 mmol/L). If a patient was in this range during hospitalization we defined that as a good glycemic control as opposite to those patients in whom we failed to achieve this target.

We performed several comparative analyses. We compared diabetic versus non diabetic patients. We also compared patients with good glycemic control versus uncontrolled patients. Based on HgbA1C and prediagnosed diabetes we subdivided the patients in five groups: three groups without known diabetes: non-diabetic (<5.6%), pre-diabetic (5.6-6.5%), newly diagnosed diabetic (≥6.5%), and two groups of pts. with known diabetes: controlled (<7%), and uncontrolled (≥7%). Cardiac event rate was analyzed as a function of these glycemic variables.

### Statistical analysis

Descriptive and comparative statistics for continuous variables with t-test (and non-parametric test for small samples), Chi square test for categorical variables (Pearson Chi square) and Fisher exact test for 2×2 tables and Odds Ratio (with Mantel-Haenszel common odds ratio), uni and multivariate logistic regression analysis for identifying the predicting variables and obtaining the ROC curves. Significance was determined at 0.05.

## Results

The study population consisted of 150 patients, 93 males and 57 females (overall mean age 62.9 ± 12.3 years) According to their HgbA1C level, glucose profile and known diabetes patients were divided in five groups: Group 0: non-diabetic patients (37.3%); Group 1: newly diagnosed pre-diabetes (24.7%); Group 2: newly diagnosed diabetes (5.3%); Group 3: known diabetes good controlled (14.0%), and Group 4: known diabetes uncontrolled (18.7%).

**Figure 1:**
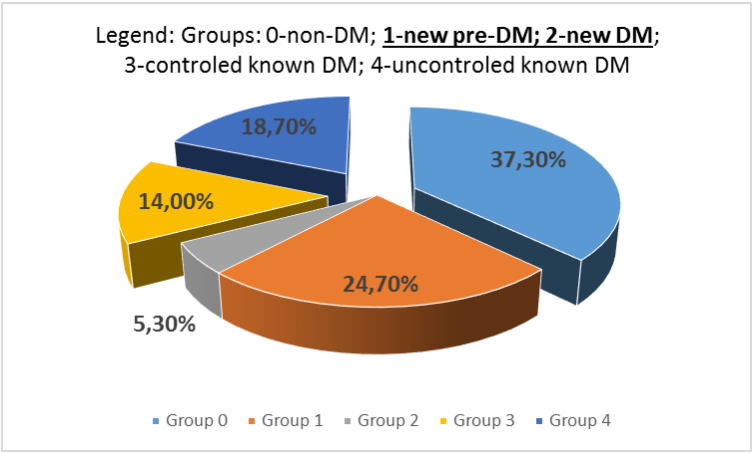
*Distribution of patients according to their diabetic status. DM indicates diabetes mellitus*.

### Baseline characteristics

Male patients predominated in our study and they were significantly younger in comparison to the females (p = 0.003). Female patients were more often hypertensive (OR 1.46; CI 1.16-4.98) and had DM (OR 1.36; CI 0.99-2.46), while males were more often smokers (OR 4.66; CI 2.29-9.45) and had positive familial history of coronary artery disease (CAD). We didn’t find gender differences in the mean ejection fraction, but reduced LV systolic function was more often present in females (p = 0.030). Also, no significant gender difference was found in CAD distribution or PCI outcome. The only significant difference in the biochemical parameters between genders was found for Hgb which was significantly lower in females (p = 0.000), but in the normal range.

When we made a comparison of these same variables between patients with and without DM, we found that non DM patients had OR of 2.1 (CI 1.16-2.40; p = 0.002) for smoking. But, there were no significant differences in LV function or in CAD distribution or PCI outcome. DM pts had significantly higher Tg levels (p = 0.041) and variables of glycemic control: HgbA1C and stress glycaemia (p = 0.000 for both variables).

The baseline characteristics of the patients as a function of the glycemic metabolism revealed that newly diagnosed diabetes was far more frequent in females (75%) and pre-diabetes predominated in males (77.6%), both groups being significantly older. Based on HbgA1C levels we identified 45 out of 101 patients (44.5%) without known DM to be diabetic (7.9%) or prediabetic (36.6%). Mean HgbA1C levels were high in newly diagnosed DM, but even higher in uncontrolled DM pts. The same was for stress glycaemia levels. The highest levels were in new DM and uncontrolled known DM, as compared with controlled known DM (p = 0.026 and p = 0.001 respectively).

**Table 1:** Baseline characteristics of ACS patients, and according to gender

Variable	Total (N)	Males (M)	Females (F)	Significance (p)
Gender	150	93 (62%)	57 (38%)	0 .000

Age	62 .9±12 .3	60 .6±11 .8	66 .7±12 .4	0 .003

HTA	85 (56 .7%)	44 (47 .3%)	41 (71 .9%)	0 .002 (OR for female gender 1 .46; CI 1 .16-1 .98; p=ns)

HLP	22 (14 .7%)	13 (14 .0%)	9 (15 .8%)	0 .468

Diabetes	49 (32 .7%)	25 (26 .9%)	24 (42 .1%)	0 .025 (OR for female gender 1 .36; CI 0 .99-2 .46; p=0 .034)

Family history	48 (32%)	34 (36 .6%)	14 (24 .6%)	0 .088 (OR for male gender 1 .77 . p=ns)

Smoking	89 (59 .3%)	68 (73 .1%)	21 (36 .8%)	0 .000 (OR for male gender 4 .66; CI 2 .29-9 .45; p=0 .000)

**Type of ACS**				

1 - APNS	24 (16%)	14 (15 .0%)	10 (17 .5%)	0 .704
2 - NSTEMI	12 (8%)	8 (8 .6%)	6 (10 .5%)	
3 - STEMI inferior	57 (38%)	35 (37 .6%)	22 (38 .6%)	
4 - STEMI anterior	57 (38%)	38 (40 .9%)	19 (33 .3%)	

**LV function**				

EF (%)	51 .3±8 .1	51 .9±8 .8	50 .2±6 .8	0 .215

LV systolic function	83 (55 .3%)	59 (63 .4%)	24 (42 .1%)	0 .030
Normal (EF >50%)	59 (39 .3%)	29 (31 .2%)	30 (52 .6%)	
Reduced (EF <50%)	8 (5 .3%)	5 (5 .4%)	3 (5 .3%)	
Severely reduced (<30%)				

LV diastolic dysfunction	65 (43 .3%)	41 (44 .1%)	24 (42 .1%)	0 .474

**CA**	132 (88%)	83 (89 .2%)	49 (85 .9%)	0 .184	
**SINTAX score**	15 .6±8 .1	15 .2±8 .4	16 .2±7 .4	0 .470	

TIMI score before treatment				
0	63 (47 .7%)	38 (45 .8%)	25 (51 .0%)	0 .184
1	7 (5 .3%)	2 (2 .4%)	5 (10 .2%)	
2	7 (5 .3%)	5 (6 .0%)	2 (4 .1%)	
3	55 (41 .7%)	38 (45 .8%)	17 (34 .7%)	

TIMI score after treatment				
0	8 (6 .1%)	5 (6 .0%)	3 (6 .1%)	0 .863
1				
2	4 (3 .0%)	2 (2 .4%)	2 (4 .1%)	
3	120 (90 .9%)	76 (91 .6$)	44 (89 .8%)	

Blood analyses				
Hgb	14 .1±1 .8	14 .5±1 .6	13 .3±1 .7	0 .000
BUN	7 .1±4 .5	6 .8±4 .6	7 .6±4 .4	0 .288
Creatinine	95 .7±72.6	90 .1±41.5	104 .8±105.2	0 .229
Na	137 .5±4 .5	137 .9±3 .4	136 .7±5 .8	0 .127
K	4 .3±0 .7	4 .3±0 .6	4 .2±0 .8	0 .474

Lipid profile				
Tg	1 .8±0 .8	1 .7±0 .8	1 .8±0 .9	0 .495
HDL chol	1 .2±0 .4	1 .1±0 .4	1 .2±0 .3	0 .270
LDL chol	3 .3±1 .2	3 .3±1 .2	3 .4±1 .1	0 .585
Lp(a)	35 .0±33 .2	33 .9±34 .4	36 .9±31 .5	0 .592

Glycemic parameters				
Stress glycaemia	10 .3±6 .6 (range 4-45)	9 .5±5 .9	11 .6±7 .4	0 .061
HgbA1C	6 .4±1 .5 (range 3 .5-11 .9)	6 .2±1.3	6 .7±1.8	0 .067
		16 (21 .5%)	12 (33 .3%)	0 .352 (ns)
No glycemic control*	28 (26%)			

In-hospital CE	31 (20 .7%)	20 (21 .5%)	11 (19 .3%)	0 .458 (ns)
Malignant arrhythmia (1)	10 (6 .6%)			
Acute heart failure (2)	5 (3 .3%)			
Shock cardiogenic (3)	5 (3 .3%)			
GIT bleeding (4)	5 (3 .3%)			
CV insult (6)	4 (2 .7%)			
In-stent thrombosis (7)	2 (1 .3%)			
Cardiac death (5)	11 (7 .3%)	8 (8 .6%)	3 (5 .3%)	0 .338 (ns)

Length of hospitalization	4 .5±3 .0	4 .3±2 .7	4 .9±3 .4	0 .252 (ns)

Legend:

* glycemic control (range of glycaemia during hospitalization >10 mmoll/L; ACS-Acute Coronary Syndrome; HTA-arterial hypertension; HLP-hyperlipidemia; ACS- acute coronary syndrome; APNS-unstable angina; NSTEMI-non ST-segment elevation myocardial infarction; STEMI-ST-segment elevation myocardial infarction; EF-ejection fraction; CA-coronary angiography; CE-cardiac events; CV-cerebro-vascular; GIT-gastro-intestinal tract.

Smoking was the only risk factor that significantly differed between groups. The smallest proportion of smokers was among pts with controlled DM. Newly diagnosed DM had the worst biochemical parameters: low Hgb (p < 0.05 in comparison to all groups), high BUN and creatinine as renal function parameters. But there were no significant differences in the Lp fractions. No significant difference was found for LV function, as opposite to CAD distribution, which was found to be the worst in newly diagnosed DM pts who had worst TIMI flow before treatment, but no intergroup differences were found after the PCI procedure. The mean hospitalization time was 4.5 ± 3.0 days with the longest duration in pts with newly diagnosed DM (p = 0.035). The event rate was 20.7% during the hospital treatment with in-hospital mortality rate of 7.3%.

**Table 2:** Baseline characteristics of ACS patients in accordance of the presence of known DM

Variable	All pts	DM pts	NDM pts	Sig (p)
**Risk factors**				
HTA	85 (56 .7%)	32 (65 .3%)	53 (52 .5)	ns (OR for HTA in DM pts 1 .7; CI 0 .84-3 .45; p=ns)
HLP	22 (14 .7%)	7 (14 .3%)	15 (14 .8%)	ns
Family history	48 (32%)	17 (34 .7%)	31 (30 .7%)	ns
smoking	89 (59 .3%)	20 (40 .8%)	69 (68 .3%)	0 .001 (OR for NDM smokers is 2 .1; CI 1 .16-2 .4; p=0 .002)

**LV function**				

EF (%)	51 .3±8 .1	51 .0±7 .3	51 .4±8 .5	0 .802 (ns)

LV systolic function				
Normal (EF >50%)	83 (55 .3%)	25 (51 .0%)	58 (57 .4%)	0 .247(ns)
Reduced (EF <50%)	59 (39 .3%)	23 (46 .9%)	36 (35 .6%)	
Severely reduced (<30%)	8 (5 .3%)	1 (2 .1%)	7 (7%)	

LV diastolic dysfunction	65 (43 .3%)	21 (42 .8%)	44 (43 .6%)	0 .538 (ns)

**CA**	132 (88%)	45 (91 .8%)	87 (86 .1%)	0 .184 (ns) 0 .365(ns)
**SINTAX score**	15 .6±8 .1	16 .5±8 .5	15 .1±7 .9	

TIMI score before treatment				
0	63 (47 .7%)	23 (55 .5%)	40 (45 .9%)
1	7 (5 .3%)	2 (4 .4%)	5 (5 .7%)	0 .862 (ns)
2	7 (5 .3%)	3 (6 .7%)	4 (4 .6%)	
3	55 (41 .7%)	17 (37 .8%)	38 (43 .7%)	

TIMI score after treatment				
0	8 (6 .1%)	5 (11 .1%)	3 (3 .4%)	0 .162 (ns)
1				
2	4 (3 .0%)	2 (4 .4%)	2 (2 .3%)	
3	120 (90 .9%)	38 (84 .4%)	82 (94 .4%)	

Blood analyses				
Hgb	14 .1±1.8	14 .0±1.6	14 .1±1.9	0 .678 (ns)
BUN	7 .1±4 .5	7 .5±4 .6	6 .9±4 .5	0 .455 (ns)
Creatinine	95 .7±72 .6	105 .3±106 .7	91 .0±48 .2	0 .260 (ns)
Na	137 .5±4 .5	135 .2±5 .4	138 .5±3 .6	0 .000
K	4 .3±0 .7	4 .4±0 .7	4 .2±0 .6	0 .103 (ns)

Lipid profile				
Tg	1 .8±0.8	1 .9±0.9	1 .7±0.8	0 .041
HDL chol	1 .2±0 .4	1 .1±0 .2	1 .2±0 .4	0 .483 (ns)
LDL chol	3 .3±1 .2	3 .4±1 .1	3 .3±1 .2	0 .615 (ns)
Lp(a)	35 .0±33 .2	38 .8±35 .3	33 .2±32 .3	0 .334 (ns)

Glycemic parameters				
Stress glycaemia	10 .3±6 .6	14 .8±7 .9	8 .2±4 .5	0 .000
HgbA1C	6 .4±1 .5	8 .2±4 .5	5 .7±0 .8	0 .000

In-hospital CE	31 (20 .7%)	11 (22 .4%)	20 (19 .8%)	0 .431 (ns)
Cardiac death (5)	11 (7 .3%)	5 (10 .2%)	6 (5 .9)	0 .266 (ns)

Length of hospitalization	4 .5±3 .0	4 .4±2 .8	4 .5±3 .1	0 .881 (ns)

**Figure 2:**
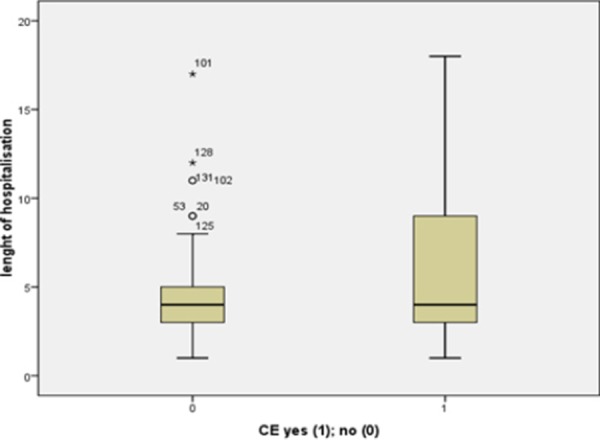
*Length of hospitalization as a function of in-hospital morbidity*.

Although the number of patients in each group was small and the groups were unequal, the highest death rate was registered among patients with newly diagnosed and uncontrolled known DM. When we analyzed the duration of hospital treatment as a function of in-hospital morbidity and mortality we registered a significant difference (4.2 ± 2.5 for pts without, versus 5.7 ± 4.2 for pts with, p = 0.009), as shown in Figure 2.

**Table 3:** Baseline characteristics of ACS patients according to glycose metabolism

Variable						
HgbA1C (%)	0-Non DM	1-Newly diagnosed pre-DM	2-Newly diagnosed DM	3- Known DM good controlled	Known DM uncontrolled	Sig (p) Pearson Chi Square or ANOVA and Post hoc Tukey

N	56 (37 .3%)	37 (24 .7%)	8 (5 .3%)	21 (14 .0%)	28 (18 .7%)	0 .000

HgbA1C	5 .2±0 .5	5 .9±0 .2	7 .6±1 .1	6 .2±0 .5	9 .0±1 .2	0 .000 for all except 1 vs 3 p=ns

Stress glycaemia	7 .1±2 .2	7 .8±2 .9	17 .7±9 .9	11 .3±4 .7	17 .4±8 .8	0 .000 for 0 and 1 vs 2 .4; 0 .012 for 0 vs 3 0 .026 for 2 vs 3 0 .001 for 3vs 4

Age	59 .3±13 .6	65 .4±11 .9	71 .7±8 .7	65 .2±9 .2	62 .5±11 .5	0 .023 0 .054 for 0 vs 2

Gender						
•	15(26 .8%)	12 (32 .4%)	6 (75%)	13 (61 .9%)	11 (39 .3%)	0 .010
•	41(73 .2%)	25 (77 .6%)	2 (25%)	8 (38 .1%)	17 60 .7%)

Smoking % in the group	46 (82%)	20 (54%)	3 (37 .5%)	7 (33 .3%)	13 (46 .4%)	0 .000

Diastolic dysfunction	22(14 .7%)	18 (12%)	4 (2 .7%)	9 (6%)	12 (8 .5%)	0 .917 (ns)

Hgb	14 .4±1 .4	14 .2±1 .9	11 .9±2 .9	14 .1±1 .5	13 .9±1 .7	0 .008 0 vs 2 0 .003 1 vs 2 0 .009 2 vs 3 0 .023 3 vs 4 0 .050

BUN	6 .3±4 .1	6 .9±4 .2	11 .1±6 .2	7 .3±3 .1	7 .6±5 .5	0 .074
Creatinine	90 .7±56 .5	86 .7±29 .9	113 .6±52 .6	124 .0±152 .4*	91 .3±50 .6	0 vs 2 0 .040 0 .332 (ns)
Na	138 .5±3 .1	138 .1±3 .8	140 .7±4 .9	135 .0±7 .1	135 .3±3 .9	0 .000 0 vs 3 0 .014 0 vs 4 0 .012 2 vs 3 0 .014 2 vs 4 0 .016
K	4 .3±0 .6	4 .1±0 .5	3 .9±0 .9	4 .3±0 .6	4 .5±0 .8	0 .094 (ns)

LP profile						
Tg	1 .7±0 .8	1 .6±0 .7	2 .0±1 .1	1 .8±0 .8	2 .1±1 .1	0 .128 (ns)
HDL chol	1.1±0 .5	1.3±0 .4	1.2±0 .3	1.2±0 .2	1.1±0 .3	0 .181 (ns)
LDL chol	3 .2±1 .3	3 .4±1 .1	3 .5±0 .7	3 .3±1 .1	3 .5±1 .1	0 .897 (ns)
Lp(a)	37 .2±32 .9	23 .9±18 .4	47 .9±61 .38	32 .3±19 .9	43 .7±43 .2	0 .107 (ns)

EF (%)	51 .7±9 .0	52 .1±7 .2	45 .6±9 .0	51 .7±6 .9	51 .3±8 .1	0 .314 (ns)

Syntax score	14 .0±7 .9	15 .9±7 .7	20 .8±5 .9	15 .3±9 .3	17 .3±7 .9	0 .261 (ns)

TIMI flow before treatment	1 .8±1 .4	1 .1±1 .4	0 .2±0 .5	1 .6±1 .5	1 .1±1 .4	0 .031

TIMI flow after treatment	2 .9±0 .4	2 .9±0 .5	2 .4±1 .3	2 .6±0 .9	2 .6±0 .9	0 .214

CE	11 (19 .63%)	5 (13 .5%)	4 (50%)	2 (9 .5%)	9 (32 .1%)	0 .056
CD (in the group)	4 (7 .1%)	0	2 (25%)	0	5 (17 .8%)	0 .012

Hospitalization (days)	4 .5±2 .7	3 .9±2 .8	7 .2±5 .3	4 .1±2 .3	4 .7±3 .1	0 .072 1 vs 2 0 .035

Legend: 0-non diabetic patients HgbA1C ≤ 5,7%; 1-newly diagnosed prediabetes HgbA1C 5,7 to 6,5%; 2-newly diagnosed diabetes HgbA1C >6,5%; 3-known diabetes good controlled (HgbA1C ≤7%); 4-known diabetes uncontrolled (HgbA1C >7%); BUN-blood urea; LP-lipoprotein; CE-cardiac event; CD-cardiac death.

* there were 3 pts. on chronic hemodialysis treatment what is the reason for higher mean and high SD.

Aiming to identify predictors of cardiac events (CE) and cardiac death (CD), and to define the role of glycemic metabolism variables as CE predictors, we performed a univariate binary logistic regression (for categorical variables) and a linear analysis (for continuous variables) and identified advanced age and smoking, Hgb (lower), BUN (higher), creatinine (higher) and HDL cholesterol of the risk factors, reduced LV systolic function and angiographic variables: Syntax score and TIMI flow. We also identified stress glycaemia, HgbA1C (as a definer of newly diagnosed and uncontrolled known DM) and established glycoregulation as significant predictors of CE and CD.

In an attempt to identify independent predictors of cardiac events we performed a multivariate logistic regression analysis using the variables found as predictors in the univariate analysis. Variables included in the prediction model were: age, cigarette smoking, EF as a continuous and a categorical variable (three categories: 0-normal, 1-mildly/moderately reduced; 2-severely reduced), TIMI flow and Syntax score, Hgb, BUN, creatinine, stress glycaemia, glucoregulation, and HDL cholesterol. We applied a backward stepwise conditional model with Chi square 38.675; sig 0.001, correct prediction 88, 6%, and at step 10 we identified four independent predictors: smoking (Exp(B) 5.945; CI 1.79-19.66); p = 0.004), EF (%) (beta -0.089; p = 0.007), HDL chol (-3.179; p = 0.016), and glycoregulation (yes) (Exp(B) 0.324 (CI 0.08-1.19; p = 0.060).

**Table 4:** Univariate predictors of in-hospital cardiac events (binary logistic regression)

Variable	Chi square	sig	Wald	Exp (B)	sig
Age	6 .819	0 .009	Beta .045		0 .012
Smoking	4 .825	0 .028	4 .742	.409	0 .029
Stress glycaemia	9 .574	0 .002	Beta .087		0 .004
HgbA1C	8 .596	0 .072			
• Group 2			3 .114	.330	0 .078
• Group 4			3 .158	.222	0 .076
HgbA1C (>6 .5%)	7 .496	0 .006	Beta -1.161		0 .006
Glycoregulation*	6 .474	0 .011	6 .803	.303	0 .009
Hgb	14 .141	0 .000	Beta -.425		0 .000
BUN	25 .548	0 .000	Beta .242		0 .000
Creatinine	4 .962	0 .026	Beta .005		0 .037
HDL cholesterol	9 .443	0 .002	Beta -2.444		0 .007
Reduced EF (<50%)	14 .978	0 .001	7 .847	.107	0 .005
EF (%)	9 .677	0 .002	Beta -.076		0 .003
TIMI flow before PCI	0 .113	0 .028			
• TIMI 0			6 .486	5 .417	0 .011
• TIMI 1			3 .562	6 .933	0 .059
Syntax score	5 .030	0 .025	Beta .065		0 .026

Legend: glycoregulation (Gl range 6-10mmol/L); BUN-blood urea; PCI-percutany coronary intervention

We applied the same model for the prediction of cardiac death only without angiographic variables because 4 cardiac deaths occurred in patients who did not underwent coronary angiography (Chi square 47.419; sig 0.000, correct prediction 94.7%) at step 6 we identified three independent predictors: EF (beta -0.368; p = 0.014), BUN (beta 0.267; p = 0.002), and stress glycaemia (beta 0.146; p = 0.060).

During the analysis of the performance capability of stress glycaemia to identify/predict cardiac events and cardiac deaths we found that both in-hospital morbidity and mortality in ACS patients are highly predictable and defined by the stress glycaemia level.

**Figure 3:**
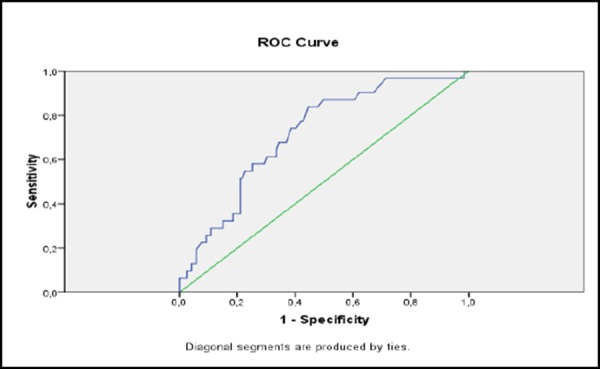
*Prediction of CE with stress glycaemia (ROC curve)*.

The classification of performance capability of stress glycaemia on cardiac events presented with a ROC curve found an area under the curve of 0.716 and p = 0.000.

The classification of performance capability of stress glycaemia on cardiac death presented with a ROC curve found an area under the curve of 0.850 and p = 0.000.

**Figure 4:**
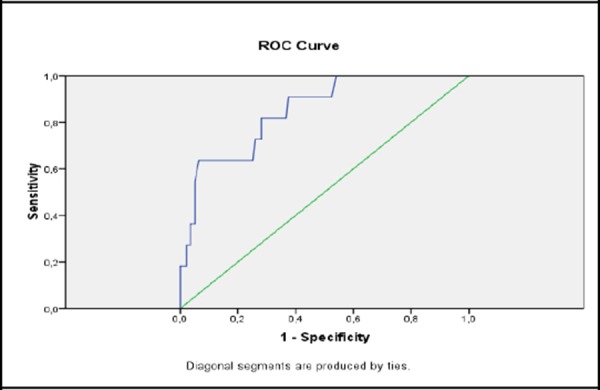
*Prediction of CD with stress glycaemia (ROC curve)*.

## Discussion

Prevalence of newly diagnosed diabetes

In our study we found only 5% of the patients to present with newly diagnosed diabetes and nearly 25% of pre-diabetes, which means that nearly one third of the patients with acute coronary syndrome had previously unrecognized impaired glycose metabolism.

Abdullatef in his study of the Qatarian population with acute coronary syndrome reports a very high prevalence of newly diagnosed diabetes in 21%, pre-diabetes in 14%, and stress hyperglycemia in 10% of the patients, predominantly males and elderly. Deane and Horowitz reported that the prevalence of newly diagnosed diabetes in critically ill patients is around 10-15%. Gardner reported that admition hyperglycemia was found in 41% of the elderly patients with acute coronary syndrome [2, 3, 8].

Elderly patients and males predominated. as found in the majority of the studies. But it seems that in terms of newly diagnosed diabetes, the prevalence in our study was smaller, for what we don’t have any logical explanation, except the small study sample. Similar prevalence of 5% reports Chih in the Australian cohort of patients with acute coronary syndrome [9].

One third of the patients were with known diabetes. Similar numbers have been reported for hospitalized patients because of acute coronary syndrome and 10-15% for critically ill patients treated in ICU [3].

### Hyperglycemia and newly diagnosed diabetes and in-hospital morbidity/mortality

The association of diabetes with increased risk of complications in patients with acute coronary syndrome is a well-known fact. But, less established are the facts that stress hyperglycemia is caring out an increased risk of complications, not only in diabetic but also in patients with previously unknown diabetes. Simon et co-workers reported a positive linear association between the degree of hyperglycemia and the mortality in patients with acute coronary syndrome, independently of the presence of confirmed diabetes [10].

In our study we found that stress hyperglycemia was more pronounced in newly diagnosed diabetics, as compared to patients with well controlled known diabetes. The occurrence of complications was associated with stress hyperglycemia and failure to achieve good glycemic control during hospitalization, not with the presence of diabetes. Patients with newly diagnosed diabetes had the longest hospitalization time and the highest in-hospital morbidity and mortality rates. Although we had a small sample size of newly diagnosed DM patients, the death rate in these was 25% and 18% in known uncontrolled diabetes.

Hyperglycemia is an even more significant predictor of complications as compared with diabetes per se. Patients with stress hyperglycemia with no previous history of diabetes have worse clinical outcome compared to those with pre-existing diabetes with a comparable degree of hyperglycemia. The impact of hyperglycemia on the clinical outcome depends of several factors such as: the intensity of hyperglycemic response, the underlying disease, the co-morbidities, the caloric intake and the risk of infection. Patients with stress hyperglycemia had a higher mortality rate and longer hospital stay compared to those with known diabetes and those with normoglycaemia [2, 4-7, 11].

*Points to be remembered:* Routine testing for glicolizated Hgb seems reasonable in patients admitted due to ACS in all patients hospitalized because of ACS. It helps us to identify diabetic patients yet unidentified. Second, even more important goal, is to establishe a good glycemic control in both cohorts of patients, newly diagnosed and known DM, in order to decrease in-hospital complications and length of hospitalization and increase survival of patients treated because of acute coronary syndrome.

*Limitations:* One of the major limitations of the study is the small sample size. For these types of studies a bigger number of participants are needed, and of course long term follows up studies.

In conclusion, we identified high prevalence of previously unknown pre-diabetes and diabetes in ACS patients. Stress hyperglycemia and failure to achieve good glycemic control during the hospital treatment were found to be independent predictors of in-hospital morbidity and mortality.
